# A potential therapeutic approach for ulcerative colitis: targeted regulation of mitochondrial dynamics and mitophagy through phytochemicals

**DOI:** 10.3389/fimmu.2024.1506292

**Published:** 2025-01-07

**Authors:** Jianping Zhou, Yuting Xi, Ting Wu, Xiaoyu Zeng, Jun Yuan, Lei Peng, Hao Fu, Ce Zhou

**Affiliations:** ^1^ Hospital of Chengdu University of Traditional Chinese Medicine, Chengdu, China; ^2^ Zigong Hospital of Traditional Chinese Medicine, Zigong, China

**Keywords:** UC, mitochondrial dynamics, mitophagy, targeted therapy, nature products

## Abstract

Mitochondria are important organelles that regulate cellular energy and biosynthesis, as well as maintain the body’s response to environmental stress. Their dynamics and autophagy influence occurrence of cellular function, particularly under stressful conditions. They can generate reactive oxygen species (ROS) which is a major contributor to inflammatory diseases such as ulcerative colitis (UC). In this review, we discuss the key effects of mitochondrial dynamics and mitophagy on the pathogenesis of UC, with a particular focus on the cellular energy metabolism, oxidative stress, apoptosis, and immunoinflammatory activities. The therapeutic efficacy of existing drugs and phytochemicals targeting the mitochondrial pathway are discussed to reveal important insights for developing therapeutic strategies for treating UC. In addition, new molecular checkpoints with therapeutic potential are identified. We show that the integration of mitochondrial biology with the clinical aspects of UC may generate ideas for enhancing the clinical management of UC.

## 1 Introduction

Ulcerative Colitis (UC) is an inflammatory bowel disease (IBD) affecting the rectum and extending to the proximal colon parts (1). In 2023, the global prevalence of UC was estimated to be 5 million cases, with over 400 diagnoses per 100,000 people reported in North America (2–4). The development of UC involves multiple pathways including changes in genetic mutations, environmental influences, impaired gut microbiota, and imbalance in the mucosal immune system (5). The primary clinical symptoms of UC patients are blood in the stool, diarrhea, and abdominal pain, fever, dehydration, weight loss, and possibly depression, anxiety, sleep disorders, and sexual dysfunction (6–9). If untreated, UC can potentially increase the risk of colon cancer (10). Several treatments have been proposed for UC, which include 5-aminosalicylic acid (5-ASA), corticosteroids, immunosuppressants, biologics, and even surgical procedures. However, the maximum response to these treatments is estimated at 30% to 60% (11). Moreover, for patients with UC, the rate of colon resection are 12%-19% at 12 months despite treatment with sequential therapy (12). In recent year, UC has become an intractable clinical challenge due to the lack of safe and long-lasting treatment options.

The available treatments focus on alleviating symptoms, preventing complications and improving the patients’ quality of life (13). Evidence from previous studies has shown that the intestinal epithelial barrier is impaired in UC patients, accompanied with dysbiosis of gut microbiota, and a dysregulated immune response (4). Furthermore, mucosal destruction and oxidative-antioxidant imbalance have been recognized as the primary factors influencing the recurrence of UC (14). Oxidative stress (OS), mediated by ROS, plays a crucial role in the initiation of inflammatory response in the colon through positive feedback mechanisms (15). The mitochondria facilitate the production of ROS, which can be extremely harmful to cells at excessive levels. Uncontrolled ROS production from damaged mitochondria increases inflammatory reactions. Studies have demonstrated that mitochondria are cellular hubs for infection (16).

Roediger et al. reported that the UC can be classified as a metabolic disorder arising from mitochondrial dysfunction. For instance, impaired mitochondrial dynamics and mitophagy were detected in DSS- or TNBS-induced mouse models of enterocolitis and in UC patients (17–19). Moreover, the mitochondrial dynamics and mitophagy have been extensively investigated as important sources of disease biomarkers, including UC. Research has uncovered that, besides the environmental factors, cytokines changes in antimicrobial pathways, and autophagy form part of the pathomechanisms of UC, driven by numerous pathways (20–22). Currently, drugs such as mitochondrial fission antagonist P110 and Mdivi-1 are being investigated for their potential to treat UC, but their therapeutic efficacy is unknown and some side effects have been reported (18, 23). Phytochemicals are extracts from natural products with numerous advantages such as multi-targeting properties, few side effects and less costly (24). Several agents targeting mitochondrial dynamics and mitophagy have been explored for the management of degenerative neurological diseases, tumors, and osteoarthritis (25–27). Therefore, we aimed to discuss the alterations in mitophagy and mitochondrial dynamics in UC as reported the available studies. The treatment of UC using natural ingredients is discussed to provide new ideas for better management of UC in the future.

## 2 Mitochondrial dynamics

**Figure 1:**
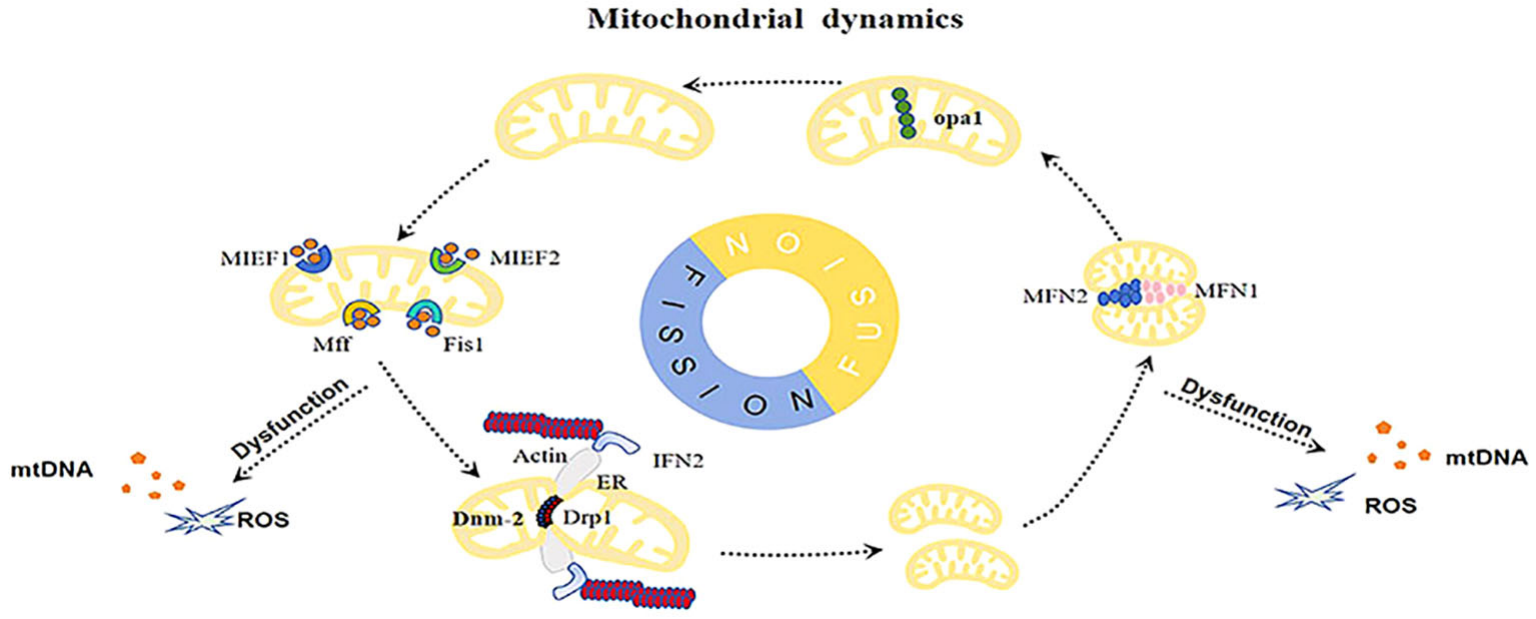
Abnormal mitochondrial dynamics increase the accumulation of damaged mitochondria, resulting in the buildup of mtDNA, ROS, and other damage-associated molecular patterns (DAMPs), inducing inflammation and cellular stress.

Mitochondrial dynamics comprises fusion, fission, and transport processes (28). Mitochondrial fission and fusion, along with their distribution along cytoskeletal trajectories, are highly coordinated mechanisms involved in the regulation of the mitochondrial network (**Figure 1**). Proper mitochondrial dynamics are driven by the biogenesis, turnover, distribution of mitochondrial DNA(mtDNA), and metabolic status (29). Studies have demonstrated that mitochondrial fission and fusion are essential components of cell survival, playing important roles in the maintenance of cellular health and disease development.

### 2.1 Mechanisms of mitochondrial fission

Mitochondrial division predominantly occurs during the S, G2, and M phases of the cell cycle (30). This process is initiated by stress stimuli and is aimed at eliminating damaged mitochondria from the cell through mitophagy. Therefore, mitochondrial division prevents the buildup of damaged mitochondria, alleviating the excessive production of ROS and the subsequent cellular stress (31).

On the other hand, mitochondrial fission is driven by the dynamin-related protein 1 (Drp1), which functions at the contractile site established by the interaction of actin and the endoplasmic reticulum (ER) (32). Phosphorylation-activated Drp1 translocates to the outer mitochondrial membrane (OMM) where it oligomerizes to form helices that drive OMM contraction and cleavage, thereby facilitating mitochondrial fission (33, 34). Given the Drp1 lacks the pleckstrin homology (PH) structural domain required for the direct interaction with the phospholipid membrane, its activity requires adaptor proteins. In mammals, four proteins regulate the recruitment of Drp1 to the OMM: fission protein 1 (Fis1), mitochondrial fission factor (Mff), mitochondrial elongation factor 1 (MIEF1), and mitochondrial elongation factor 2 (MIEF2), that serve as Drp1 receptors to improve the fission process (35). activation of fission following the enclosement of mitochondria by the ER leads to the Mff-dependent assembly of Drp1 (36). Inactivation of the Mff gene then inhibits mitochondrial recruitment of Drp1, mitochondrial and peroxisomal elongation, while Mff overexpression can fragment the network (37, 38). It has been shown that MIEF1 binds to the adenosine diphosphate (ADP) cofactor, whereas MIEF2 recruits Drp1 to the mitochondrial surface (39). Mff and MIEF1/MIEF2 function are important receptors of Drp1, which mediate it role on mitochondrial fission. Researchers have demonstrated that overexpression of Mff or MIEF is overexpressed, they significantly attract cytoplasmic Drp1 to the mitochondria, independent of hFis1, which enhances the process of mitochondrial fission (40). These receptors work together to help Drp1 divide mitochondria. In mammals, Fis1 usually plays a minor role in recruiting Drp1. However, during cell death or autophagy, Fis1 becomes more important, significantly boosting mitochondrial division. The role of Fis1 in humans is still unclear (41). Finally, the GTPase dynamin 2 (Dnm2) is transiently recruited to the ER contact site downstream of Drp1 where it facilitates the fission process (42, 43). Subsequently, Drp1 is transported back to the cytoplasm, creating a dynamic cycle between the cytoplasmic space and the mitochondrial membrane. The Drp1’s activation, movement, and oscillation between cellular compartments are tightly regulated by several post-translational modifications. These include phosphorylation, ubiquitination, sumoylation, and glycosylation. The mechanisms by which these modifications alter the functionality of Drp1 are described in details in later sections of this review (44–47).

### 2.2 Mechanisms of mitochondrial fusion

Mitochondrial fusion is commonly detected in the early S and G1 phases, and is essential to the generation of sufficient ATP needed to sustain respiration and protein synthesis (48). Moreover, the fusion modulates the exchange of materials such as mtDNA, proteins, and metabolites, enhancing the repair of damaged molecules, inhibiting phagocytosis of elongated mitochondria, which may result from site-blocking during nutrient deprivation and the induction of autophagy (48, 49).

The process of mitochondrial fusion involves the fusion of the inner mitochondrial membrane (IMM) and the OMM, occurring in multiple steps beginning with the activation of dynamin-associated GTPases, including mitofusins (MFN1 and MFN2) on the OMM, and optic atrophic protein 1 (Opa1) on the IMM. This is followed by the GTP hydrolysis-induced fusion of the OMM (50). Structurally, the MFNs are embedded in the OMM via two transmembrane regions, divided by a short loop. This structure allows the N-terminal region, containing the GTPase domain, the coiled-coil heptad repeat 1 (HR1), and the C-terminal region that harbors the HR2 domain, to orient towards the cytoplasmic side (51). This is followed by the IMM fusion, orchestrated by Opa1 in the IMM and endosomal lipid components (52). During fusion, two Opa1 proteins in the IMM interact to form oligomers that assemble into flexible helices, causing membrane swelling and bringing the two IMMs into close proximity. The nucleotide binding organizes and tightens the helix assembly, pulling the IMM closer together to initiate fusion. At the end of the fusion, the helical structure of the Opa1 oligomerization is uncoiled (53). This mechanism is crucial for Opa1 to form a helical structure by dimerizing the GTPase domain. Furthermore, the membrane-bending oligomers of Opa1 undergoes conformational changes, which retract the membrane insertion loop from the outer leaflet, causing remodeling (54). OMM fusion is usually coordinated with inner membrane fusion, but can sometimes occur independently. This phenomenon may be triggered by occurrence of mutations or reduced membrane potential which impairs the fusion of the IMM, but the OMM fusion is not affected (55).

### 2.3 Factors regulating mitochondrial dynamics

#### 2.3.1 Drp1

The function of Drp1, a key regulator of mitochondrial fission, is influenced by multiple post-translational modifications, such as phosphorylation, ubiquitination, and sumoylation. Its activity is modulated through phosphorylation at three main sites: ser616, ser637, and ser693 (56). The ser616 site is phosphorylated by protein kinase Cδ (PKCδ), Rock kinase, or Pink1, which then activates fission and promotes the binding to other fission proteins (57, 58). On the other hand, the ser637 site is phosphorylated by protein kinase A, which leads to its inactivation (59). Phosphorylation of the ser693 site by GSK3β serves to inhibit mitochondrial division (60). Furthermore, the membrane-associated E3 ligase March5 can regulate Drp1 through ubiquitination (61). Likewise, the E3 ligase Mulan, which is also membrane-bound, was reported to influence Drp1 by promoting sumoylation (62). Functionally, Drp1 that functions without involvement in nitrosylation is thought to trigger mitochondrial fission (63, 64). Ubiquitination, particularly by March5, is crucial for regulating mitochondrial fission. However, while March5 ubiquitinates Drp1, it does not affect its stability. Instead, the process of ubiquitination may affect the dynamics of the Drp1’s interaction through the mitochondrial membrane. In this context, the attachment of ubiquitin alters the subcellular transport, promotes Drp1 assembly, and modulates the mitochondrial fission (61).

#### 2.3.2 MFN1/2

MFN1/2 undergoes multiple post-translational modifications, including oxidation, ubiquitination, and phosphorylation. The redox state of specific residues in MFN can regulate its activity and promote membrane fusion by triggering oligomerization (65). During stress conditions, March5 activates the ubiquitination of acetylated MFN1, which marks it for degradation via the proteasome (66). In contrast, the histone deacetylase 6 augments MFN1-mediated mitochondrial fusion, particularly in response to oxidative stress (67). March5 specifically targets mitochondrial MFN2 for ubiquitination, promoting its oligomerization and strengthening mitochondria-ER tethering (68). In the context of cell apoptotic, the E3 ubiquitin ligase Parkin targets MFN1 for ubiquitination (69) and the activity of the deubiquitinating enzyme USP30 can reverse this process, which reactivates the mitochondrial fission process (70). Phosphorylation of MFNs can either promote or inhibit mitochondrial fusion, depending on the specific site and kinase involved. For example, ERK-mediated phosphorylation of the HR1 domain in MFN1 can suppress fusion and promote its interaction with Bak, triggering apoptosis (71). Cellular stress stimulates Jnk to phosphorylate MFN2 and the subsequent recruitment ofHuwe1 to MFN2, a critical step required for its proteasomal degradation and subsequent apoptosis activation (72).

#### 2.3.3 Opa1

Studies have shown that the Opa1 is the only dynamin-like GTPase detected within the IMM. The MFN1 is by Opa1 to enhance mitochondrial fusion (52). Similarly, the Opa1’s activity is influenced by several post-translational modifications. However, the exact mechanisms need to be clarified through further studies.

The balance between mitochondrial fusion and fission benefits cells by regulating mitochondrial shape, enabling content exchange, ensuring fair mitochondrial inheritance, preserving healthy mtDNA, and eliminating damaged mitochondria (73). These structural changes in mitochondria may lead to the development of diseases by impairing the expression levels of proteins involved in mitochondrial dynamics. Moreover, abnormal activation of the signaling pathways can alter the mitochondrial dynamics (74). Previous studied investigations have shown that bacterial, viral, and parasitic pathogens can also modify host mitochondrial dynamics upon cell infection, facilitating their proliferation, significantly influencing disease outcomes (75). The subsequent mitochondrial dysfunction may activate intracellular inflammatory signaling pathways triggering the release of inflammatory factors (76). Mitochondrial dynamics contribute to the activation of immune cells (77). Specifically, the Drp1-mediated mitochondrial fission alters the T-cell activity, making it an important regulator of diverse autoimmune inflammatory diseases (78).

## 3 Mitophagy

**Figure 2:**
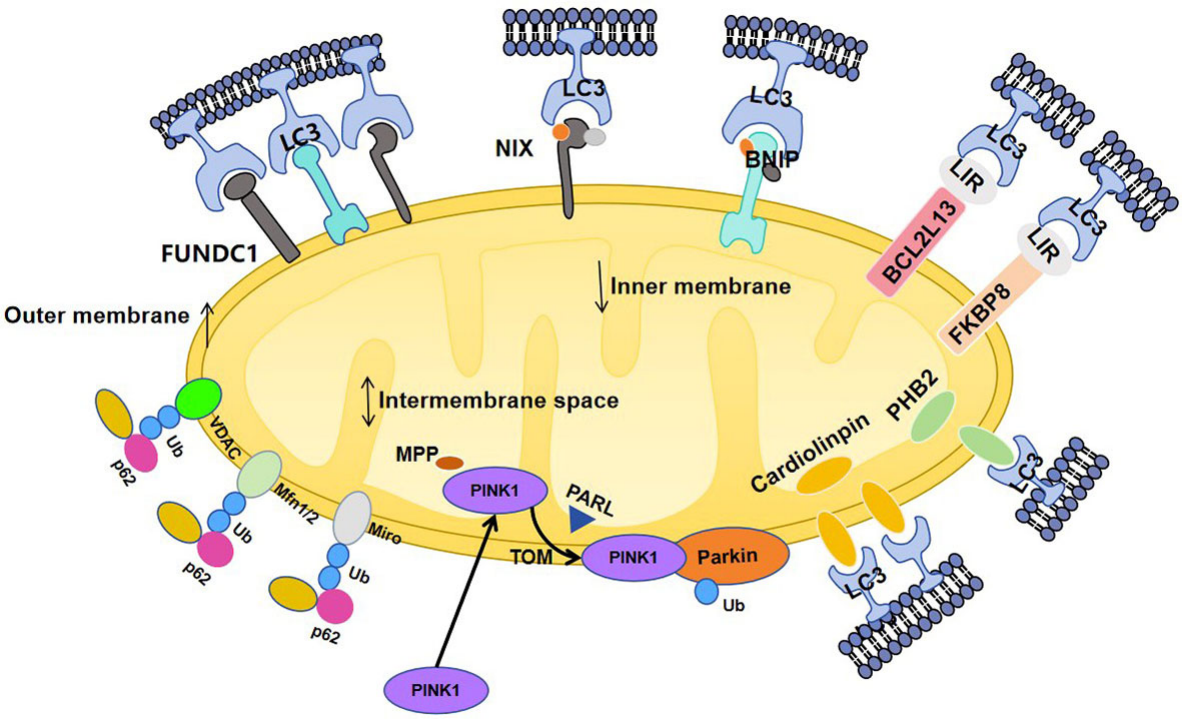
1) Pink1 is sequentially targeted to the mitochondria via a targeting sequence and is degraded by the matrix processing peptidase (MPP) and subsequently cleaved by the IMM protease progerin-associated rhodopsin-like enzyme (PARL). Pink1 accumulates on the OMM via the regulation of the enzyme translocase of the outer membrane (TOM). Accumulated pink1 is autophosphorylated and activated through autophosphorylation which then phosphorylates ubiquitin on serine 65 (Ser65), triggering the recruitment of Parkin to the mitochondrial membrane. PINK1 and its substrate, ubiquitinates, phosphorylates and activates Parkin. Specifically, polyubiquitination of Parkin substrates, such as voltage-dependent anion channel-1 (VDAC1), mfn1/2, and Miro 1, induces their degradation by the proteasome. 2) Bcl2 like 13 (BCL2L13) is the mammalian homolog of atg32. In mammalian cells, BCL2L13 facilitates mitosis independently of Parkin. Like other LC3 receptors, BCL2L13 is located on the outer mitochondrial membrane and binds to LC3 via the LIR motif. Specifically, phosphorylation of the Ser272 site enhances the binding of BCL2L13 to the LC3.FK506-binding protein 8 (FKBP8). FKBP8 is located on the outer mitochondrial membrane and stimulates mitochondrial autophagy by interacting with LC3A; 3) Cardiolipin is an inner mitochondrial membrane lipid, and PHB2 are IMM proteins, which bind to the LC3 receptor to initiate mitophagy.

Mitophagy is a selective autophagy mechanism known to maintain cellular homeostasis by eliminating damaged mitochondria. This process is initiated by mitochondrial depolarization and is especially critical for highly differentiated post-mitotic cells, which are largely dependent on aerobic metabolism (79). This process is a major regulator of mitochondrial quality control, which prevents the accumulation of potentially harmful mitochondria that initiates excessive inflammatory responses (80). Defective mitophagy can lead to inflammatory and autoimmune diseases by disrupting inflammatory cytokine secretion and immune cell function. Currently, there are two types of mitophagy pathways: the ubiquitin-dependent pathway and the non-ubiquitin-dependent pathway (**Figure 2**).

### 3.1 Pink1-Parkin

Autophagy receptors involved in the mitochondrial protein ubiquitination function through the ubiquitin-dependent mitophagy pathway, specifically the serine/threonine kinase Pink1/Parkin pathway (81). Once Pink1 is exposed on damaged mitochondria, it initiates Pink1/Parkin-dependent ubiquitination, which is often triggered by a loss of mitochondrial membrane potential (82). The expression of Pink1 is kept at low levels under physiological conditions, however, during mitochondrial damage, including mtDNA mutations, elevated mitochondria ROS (mtROS), and the buildup of misfolded proteins, the Pink1 accumulates at the OMM. This is followed by autophosphorylation and activation, as well as phosphorylation of ubiquitin on serine 65, promoting the recruiting Parkin from the cytoplasm to the mitochondrial membrane (83). Next, the Parkin is activated inducing the ubiquitination of mitochondrial proteins and triggering mitophagy (84). Even being absence in Pink1, Parkin could still be attracted to depolarized mitochondria, aiding in mitophagy. For example, HtrA2/Omi or LRRK2 can phosphorylate mitochondrial proteins or parkin itself to promote mitophagy under conditions of pink1 deficiency (85, 86). Alternatively, overexpressed or hyperactive FUNDC1 can recruit parkin to initiate autophagy (87). Among the known targets of Parkin ligase on the OMM are MFN1/2, voltage-dependent anion channel protein 1 (VDAC1), and mitochondrial GTPases (88–90). Proteomics analyses on the degradation of OMM components and the reorganization of the OMM proteome are highly advocated to increase our understanding of the process of mitophagy. Being the most abundant OMM protein, VDAC1 forms the mitochondrial pore and is a key modulator of metabolites, ions, and water transport under physiological conditions and influences mitochondrial homeostasis (91–93). The VDAC1 participates in the regulation of mitophagy, interacting with Parkin to facilitate polyubiquitination and recruitment of Parkin to induce mitophagy (94). By energizing the Pink1/Parkin mitophagy pathway, VDAC1 in conjunction with BNIP3 alters the mitophagic flux process (95). Hypoxia-induced GPCPD1 depalmitoylation has been reported to initiate mitophagy by regulating the PRKN-mediated VDAC1 ubiquitination (96). Application of the antidepressant drug sertraline was found to alter the VDAC1 protein, decrease ATP levels, activate AMPK, and inhibit the MTOR signaling pathway to induce autophagy (97).

### 3.2 NIX/BNIP3/FUNDC1 receptor-mediated

NIX and BNIP3 belong to the Bcl-2 protein family which is located on the OMM. The Bcl-2 family members on the OMM participate in the initiation of cell apoptosis, influencing the mitophagy process (98, 99). One of the mechanisms by which autophagy activates mitophagy is through the direct binding to the light chain 3 (LC3) via the BH3 structural motif. This motif functions as a molecular effector of mitochondrial membrane depolarization-induced hypoxia which promotes occurrence of mitophagy (100). NIX was reported to act as an adaptor protein that transports the components of the autophagy machinery to the mitochondria to trigger mitophagy. Another proposed model suggests that BNIP3 or NIX competes with Beclin-1 for binding to Bcl-xl. During erythropoiesis, the expression of NIX is upregulated, disrupting the Bcl-xl-Beclin-1 interaction and freeing Beclin-1 to trigger autophagy (101). Notably, the BNIP3 was demonstrated to lower the mTOR activity and regulate autophagy by increasing LC3 expression. Similar to BNIP3 and NIX, FUNDC1 is a mitochondrial junction protein induced by hypoxia that bind with LC3 via its LIR motif. Under hypoxic environments, the ULK1 kinase relocates to mitochondria, phosphorylating FUNDC1, enhancing its binding with LC3 to promote mitophagy (102).

Most researchers agree that mitophagy is closely linked to mitochondrial dynamics. Inhibiting fission with DRP1K38A or FIS1 RNAi reduces mitophagy, while overexpressing Opa1 also inhibits autophagy (103). Impaired fusion results in a reduction in IMM potential, triggering pink1 accumulation and parkin activation. However, the inverse dependence of fusion and autophagy on membrane potential makes them complementary rather than competing processes for daughter mitochondria after a fission event (104). Therefore, we suggest that mitochondrial fission is a prerequisite for mitophagy and that fusion will inhibit autophagy. However, inhibiting mitochondrial hyperfusion by silencing Drp1 or Mff does not affect mitophagy induced by Fis1 loss (105). In future, researchers should aim to explore the crosstalk between mitochondrial dynamics and mitochondrial autophagy. Moreover, several proteins involved in mitochondrial dynamics also participate in mitochondrial autophagy process. MFN1/2 is extracted from the OMM using ubiquitin-dependent chaperones and is subsequently degraded by the proteasome (106). The deletion of ubiquitination of MFN1/2 prevents the fusion of damaged mitochondria, while enhancing the fission, thereby promoting mitophagy (107). Additionally, Pink1 phosphorylates MFN2, which acts as a Parkin receptor to eliminate impaired mitochondria (108). However, it is unclear whether the OMM proteins coordinate both mitochondrial fission and mitophagy. The precise interplay between mitochondrial dynamics and autophagy requires further investigation.

## 4 Relationship between mitochondrial dynamics and UC

**Figure 3:**
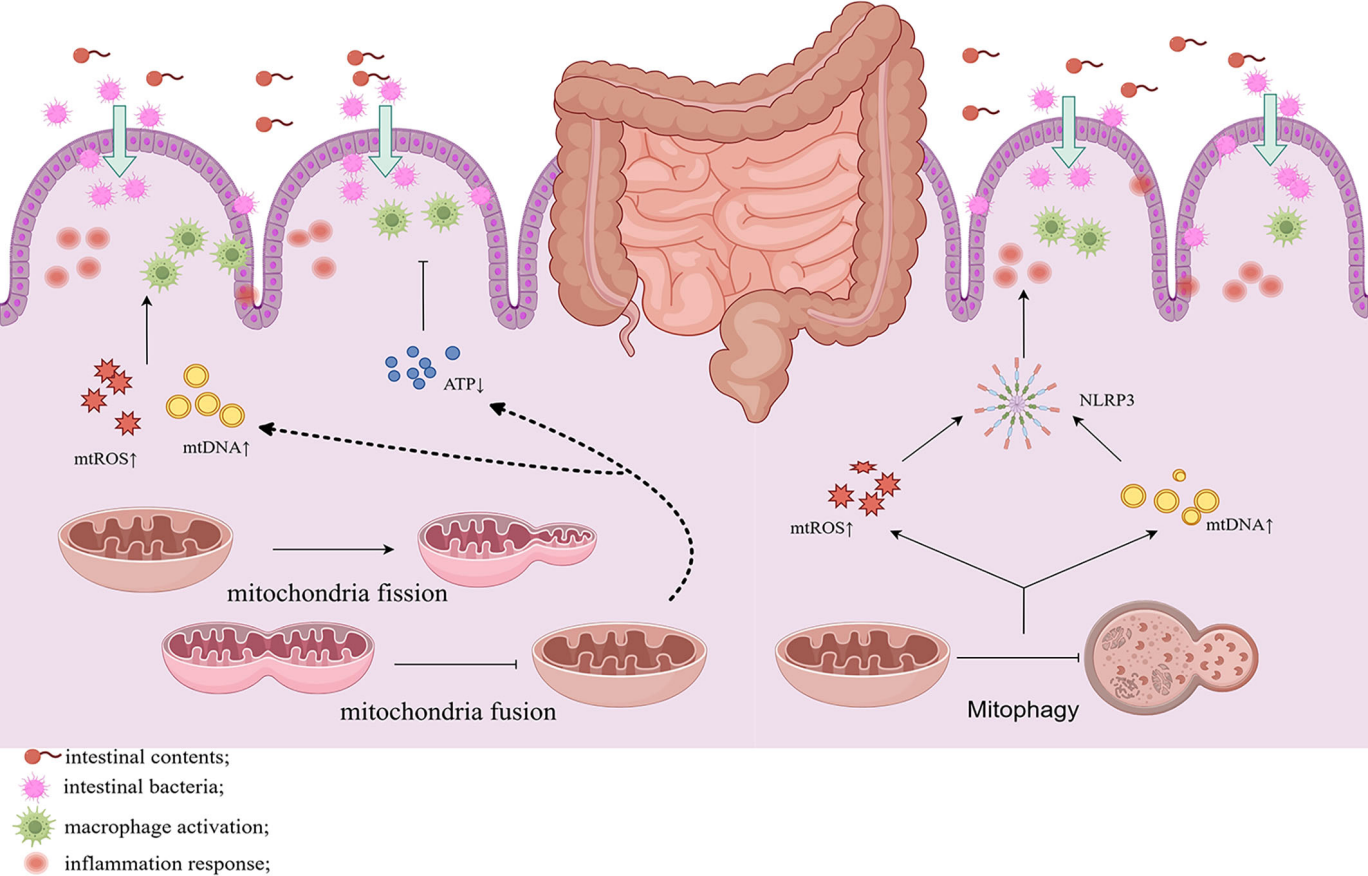
Altered mitochondrial dynamics of the intestinal cells in UC induces mitochondrial fission and inhibits fusion while attenuating mitochondrial autophagy. This results in the reduction of ATP production and promotes the release of mtDNA, mtROS, and activation of NLRP3 inflammasome, leading to inflammation and disruption of the intestinal barrier. Chronic inflammation and gut barrier disruption also activate mitochondrial fission and reduces autophagy, establishing a vicious cycle.

Previous studies have demonstrated changes in GTPase mRNA expression, such as Drp1, Opa1, and mitophagy in UC. Mancini et al. reported that in DSS-versus DNBS-treated mice with intestinal inflammation, the mRNA levels of Drp1 and Fis1 were elevated, triggering excessive mitochondrial fission (18). The expression levels of MFN1, MFN2, and Opa1 proteins were found to be downregulated in the intestinal epithelial cells of UC patients and DSS-induced mice, which weakens the mitochondrial fusion capacity and triggers mitochondrial dysfunction (109). Restoring the expression of these proteins may improve mitochondrial function in UC. In UC mice, mitochondria in the subnuclear region of inflammatory cells appear swollen and fragmented. A strong correlation exists between inflammation and the mitochondrial network disruption in colitis. The mitochondrial network is also disrupted in non-inflamed colonic regions, suggesting that the mitochondrial structure may be an early event in UC (17). Excessive mitochondrial fission and reduced fusion can stimulate the development of UC by altering energy metabolism, oxidative stress, and apoptosis (**Figure 3**).

### 4.1 Changes in energy metabolism

As early as 1980, Roediger et al. postulated that IBD, particularly UC, may be an energy-deficient disease (19). Since then, several studies have demonstrated that patients with UC exhibited reduced mitochondrial function, including structural abnormalities, mtDNA mutations, reduced electron transport chain activity, decreased oxidative phosphorylation, and lower ATP concentrations (110–113). Mitochondrial dysfunction and imbalance in dynamics are often accompanied with impaired energy metabolism, which triggers inflammation and gastrointestinal symptoms (114). The colonic epithelial cells consume a high amount of energy to execute various processes, including the electrolyte exchange in the intestinal epithelium, glycoprotein synthesis in the mucus layer, lipid synthesis in cell membranes, structural protein synthesis, and detoxification. Therefore, energy deprivation may impair these processes, causing epithelial cell atrophy in the short term and colonic mucosal barrier damage in the long-run, leading to the development of UC (115, 116). Compared to healthy donors, oxidative phosphorylation metabolism in UC patients shifts towards glycolysis as a strategy to compensate for ATP production defects. This increases the cellular levels of lactate acid within colonic cells, whereas bacteria in the intestinal lumen consume epithelial oxygen, inducing ecological dysregulation which promotes inflammation (117). Cells with inflammatory colitis may use glycolysis to produce ATP as an adaptive response to butyrate transport and oxidative stress. A reduction in ATP levels induce detrimental effects on the actin cytoskeleton, whereas actin irregularities may impair the localization and function of cellular junctions, increased gaps between cells (118, 119). Lan A et al. demonstrated that in DSS-induced enterocolitis mouse model, the ATP activity was enhanced, and the expression of energy-dependent differentiation markers was upregulated. This was accompanied by enhanced epithelial repair, requiring large amounts of energy to enhance the structural and functional recovery of the colonic mucosa. This process has been linked to the increase in expression levels of regulators of mitochondrial mass and biogenesis, such as Sirt3, FoxO3, and PGCα. It is also characterized by enhanced fusion, as evidenced by the upregulated Mfn2 gene expression and good mitochondrial morphology (120). In previous studies, creatine supplementation or administration of energy sensors improved intestinal epithelial repair. Therefore, strategies for promoting cellular energy metabolism via modulating mitochondrial dynamics and intestinal barrier are potential treatments for UC (121, 122).

### 4.2 Increased oxidative stress

Impaired mitochondrial dynamics have been linked to the development of oxidative stress in UC patients. Mitochondrial aerobic metabolism promotes the proliferation and differentiation of intestinal stem cells within colonic crypts and supports the synthesis of intestinal epithelial cells (123). However, excessive production of ROS may occur in damaged mitochondria. Under normal conditions, mitochondria exhibit a well-coordinated self-repair mechanism. However, in cases of a dynamics imbalance, oxidative stress levels increase within the cells, triggering an inflammatory response (124). Excessive mitochondrial fission reduces energy production and leads to the generation of oxidative stress, increasing mitochondrial fission, and reducing cell viability (125). Mitochondrial fusion may trigger the mixing of mitochondrial contents, including DNA and respiratory chain complexes, which are involved in the repair of damaged mitochondria and reduce localized oxidative stress levels. However, a decrease in mitochondrial fusion may increase the development of oxidative stress (126). Studies have shown that gut microbiota and their metabolites participate in this process. Colic acid, a major biofilm component of *Escherichia coli* (*E. coli*), increases mitochondrial fission in enterocytes in a Drp1-dependent manner, enhancing the stress-activated transcription factor-mediated unfolded protein response (UPR) in response to mitochondrial stress (127). DSS can cause damage to the intestinal epithelial respiration and stimulate the mitochondrial complexes I, II, and IV, leading to the accumulation of damaged mitochondria and ROS, both of which cause cellular damage (128).

The mitochondrial chaperone protein Prohibitin 1 (PHB1), an endomembrane protein, regulates Opa1-mediated fusion within the IMM (129). Mitochondrial dysfunction in intestinal epithelial cells, induced by PHB1 deletion was found to trigger spontaneous ileitis in mice (130, 131). In the study, it was observed that epithelial cells showed mitochondrial dysfunction, crypt cell death, and Paneth cell abnormalities, which are also observed in the intestinal epithelium of patients with IBD. Treatment with the mitochondria-targeted antioxidant Mito-Tempo ameliorated the Paneth cell defects and reduced intestinal epithelial inflammation in mice lacking PHB1. The role of Paneth cell mitochondrial impairment in the development of ileitis has been documented in multiple studies on PHB1-deficient mice (131).

### 4.3 Cell apoptosis

The mitochondrial dynamics have been implicated in the regulation of apoptosis. Excessive mitochondrial fission can induce morpho-functional changes in mitochondria, which triggers the production of intrinsic apoptotic signals. Studies have demonstrated that inhibition of mitochondrial fusion stimulates that intracellular stress response and enhances apoptosis (132). The pathogenic *E. coli* secreted effector proteins, mitochondria-associated protein(Map), and *E. coli* secreted protein (EapF) are within the mitochondrial matrix. Map induces mitochondrial fission, while EspF promotes the permeabilization of the mitochondrial outer membrane (MOMP) in the OMM (133). Changes in mitochondrial membrane permeabilization may be accompanied by the release of pro-apoptotic factors, such as cytochrome c, which activate the apoptotic signaling pathways (134). Mitochondrial fission resulting from MFN deficiency in HeLa cells protects against *Lactobacillus monocytogenes*, suggesting that *Listeria hemolysin O* can disrupt mitochondrial dynamics and promote apoptosis via disrupting calcium influx. In multiple cell types, enhanced mitochondrial fusion inhibits the propagation of apoptotic signals, delaying cellular apoptosis (135). This phenomenon provides a potential alternative approach for treating UC.

In neurological diseases, dysregulation of Drp1 disrupts mitochondrial dynamics, stimulating inflammation and exacerbating disease severity (136). In pancreatic islet inflammatory cells, NF-κB activation was reported to increase Opa1 expression, while interleukin-6 (IL-6) was found to increase Fis1 and downregulate MFN2, enhancing disease progression (137). In colitis, abnormal mitochondrial dynamics stimulate multiple inflammatory pathways, such as the NF-κB pathway, which activated the inflammatory response. Mitochondrial fission can also trigger apoptosis, compromising intestinal barrier function and promoting cellular inflammation. In future, researchers should explore the role of mitochondrial dynamics in UC to identify robust therapeutic disease.

## 5 Relationship between mitophagy and UC

Mitophagy provides an important mechanism for eliminating damaged mitochondria to maintain normal mitochondrial function. Excessive mitochondrial damage and mitophagy levels have been detected in the intestinal tissues of UC patients, which are correlated with high disease severity (138). In epithelial tissues of DSS-induced enterocolitis mice, the number of damaged mitochondria, autophagy and mtROS were recorded, which further activated the NLRP3 inflammasomes to promote inflammation (139). This indicates that the role of mitophagy in apoptosis is bidirectional. Excessive mitophagy can trigger energy depletion, thereby inducing apoptosis, whereas, suppression of mitophagy may potentially prevent the clearance of damaged mitochondria, increasing the generation of ROS levels and inflammation.

### 5.1 Autophagy gene and development of UC

Genome-wide association studies (GWAS) have identified several autophagy genes, including Atg16L1, Lrrk2, and Irgm, which influence the genetic susceptibility of IBD (140–142). Atg16L1 is a critical component of the mitophagy machinery involved in the regulation of immune responses and inflammation. It is part of the complex that cleaves the ubiquitin-like protein LC3 via a lipolytic mechanism, promoting autophagosome formation and activity. The Atg16L1 risk variant (Atg16L1T300A), was found to influence the risk of IBD, creating a caspase cleavage site that weakens the stability of the protein, thereby diminishing autophagy, particularly in the presence of TNF-α (143). Irgm, a human immune-associated GTPase, translocates to mitochondria, where it regulates mitochondrial division and induces mitophagy (144). Irgm1, a homologue of Irgm, participated in the development of intestinal inflammation in mice. Irgm1 knockout mice exhibited increased severity of inflammation following exposure to DSS. Mice with DSS knockout exhibited significant disruptions in Paneth cell positioning and granule structure, which contributed to mitophagy and autophagy impairment within the Irgm1-deficient enterocytes, including Paneth cells. These findings demonstrate that Irgm1 regulates acute inflammatory response in mouse intestines, probably by regulating autophagy, which modulates the normal Paneth cell function (145). Lrrk2 is a multifunctional protein with kinase and GTPase activity; with mutations in the Lrrk2 gene found to decrease mitochondrial numbers, impaired mitochondrial dynamics, and inhibition of the mitophagy pathway (146). In individuals with UC, the expression level of Lrrk2 were upregulated in peripheral blood samples. In preclinical animal models of UC, Lrrk2 knockout ameliorated the progression of DSS-induced enterocolitis, which was accompanied with the transition of the intestinal macrophages (MФs) to alternatively activated macrophages, promoting probiotic colonization and attenuating the onset and progression of colitis (147). This indicates that Lrrk2 plays a crucial role in the development of UC.

### 5.2 Mitophagy and immune inflammation response in UC

Mitophagy influences the innate and adaptive immune responses, thereby maintaining intestinal homeostasis (148). In previous studies, most studies focused on the intestinal immunity of macrophages and T cells. In UC patients and mice with DSS-induced colitis, the mitophagy of intestinal macrophages was significantly decreased. It was found that the expression of proteins associated with the Pink1/Parkin and NIX-mediated autophagy pathways was decreased. This was accompanied by the accumulation of mtROS and mtDNA, which were then released into the cytoplasm, activating the NLRP3 inflammasome and enhancing the maturation of IL-18 and IL-1β (149, 150). In a mouse model of T-cell metastatic colitis, the expression the Th1 transcription factor TBX21 and Th1 cells in the lamina propria of Pink1 knockout (Pink1KO) mice was higher compared to the levels of naïve T cells. Treatment with the Urolithin A (UA), a mitophagy agonist, inhibited the Th1 differentiation, reducing the formation of IFN-γ and IL-17, which suppresses the levels of the associated inflammatory response (151). This finding suggests that inhibiting inflammation via enhancing mitophagy may be an attractive strategy for alleviating enteritis symptoms. Several cytokines have been reported to modulate mitophagy. IL-10, a key anti-inflammatory factor, inhibits the metabolic shifts associated with inflammatory stimuli in macrophages, while maintaining mitochondrial integrity and function by inhibiting mTOR (152).

Mitochondria are important regulators of the innate immune signaling, with several immune effectors clustered on the OMM (153). Among then, MAVS which targets the OMM through its C-terminal transmembrane domain was demonstrated to activate the downstream signaling pathways via NF-κB to regulate IFN production, and its activation is modulated by mitochondrial dynamics (154, 155). Furthermore, studies have shown that depletion of autophagy proteins, such as LC3B and Beclin 1, results in the accumulation of damaged mitochondria and the translocation of mtDNA into the cytoplasm, activating inflammatory gene transcription. The activities of TOMM20 and HSP60, proteins forming part of the OMM and mitochondrial matrix, respectively, negatively correlate with autophagy. In LPS-treated UC mouse models, MODE-K cells and colonic tissues displayed increased expression of p62, TOMM20, and HSP60, causing autophagy inhibition and enhancing inflammation (156). The absence of Nix/BNIP3L-mediated mitophagy during PHB1 protein deficiency causing excessive production of ROS and activation of inflammation, which suggests that mitochondrial dysfunction may impair mitophagy (157). Studies investigating the effect of polystyrene nanoplastics (PS-NPs) on enterocolitis development revealed that the buildup of these plastics in mitochondria induced mitochondrial stress, initiating Pink1/Parkin-mediated mitophagy. Additionally, LC3 dots in Caco-2 cells co-localized with mitotracker-labeled mitochondria, indicating the initiation of mitophagy (158).

## 6 Current studies of potential therapeutic approaches to modulate both mitochondrial dynamics and mitophagy in UC

The recent advancements in research investigating potential strategies for modulating mitochondrial dynamics and mitophagy in UC have achieved significant progress. Despite the advances in UC treatment, many patients show poor response to biological therapies (159). Proper mitochondrial fission, fusion, and mitophagy are essential for mitochondrial health. Inhibition of excessive fission has been shown to enhance mitophagy with beneficial effects in animal models of myocardial infarction, pulmonary hypertension, ischemia-reperfusion injury, multiple sclerosis, and Huntington’s disease (160–164). Modulating this mechanism may help to control mitochondrial dysfunction in colitis. Pharmacological or natural products have been proposed to restore mitophagy homeostasis, promoting the clearance of irreversibly dysfunctional mitochondria, making them promising therapeutic approaches for UC.

### 6.1 Mitochondria-targeted therapy for UC

Suppressing excessive mitochondrial fission has been shown to accelerate mucosal healing in mice. Patients with UC exhibit increased mitochondrial fission, along with higher butyrate exposure in the environment surrounding colonic stem cells. Excessive mitochondrial fission inhibits stem cell proliferation by disrupting butyrate metabolism in colonic organoids. Mechanistically, results from various enzyme activity assays in colonoids have shown that excessive fission delays mucosal repair by increasing ROS leading to the inhibition of mitochondrial acetoacetyl CoA thiolase activity, which impairs butyrate metabolism. Therefore, the failure of antibiotics to promote mucosal healing in mice was reversed following the treatment of the mitochondrial fission antagonist P110 and exogenous butyrate (165). Further findings show that P110 suppresses excessive fission by blocking Drp1 binding to Fis1, significantly alleviating intestinal inflammation and reducing UC symptoms (18). Unfortunately, few drugs effectively inhibit mitochondrial fission *in vivo*. Mdivi-1 is one such drug, targeting Drp1, but it may induce unintended side effects on oxidative phosphorylation and ROS levels (23). Leflunomide, an antirheumatic drug that inhibits pyrimidine synthesis has been found to mitigate mitochondrial fission by enhancing fusion to alleviate the associated symptoms (166). In addition, Chen W and colleagues described several chemotherapeutic agents that target different mechanisms of mitochondrial fission and fusion. However, evidence-based data to support the therapeutic benefits of these agents in the treatment of UC (50).

### 6.2 Natural products on UC-related tissues through mitophagy

Natural products, such as curcumin, can treat diseases by targeting mitochondrial function. For instance, curcumin protects cartilage in osteoarthritis by stimulating AMPK/PINK1/Parkin-mediated mitophagy (167). Berberine protects glomerular podocytes by inhibiting Drp1-mediated mitochondrial fission and dysfunction (168). Natural products can regulate mitophagy making them potential treatments for UC-associated mitochondrial dysfunction (**Table 1**). To assess the therapeutic efficacy of these natural products and their potential side effects on tissues or cells, UC-associated models and *in vitro* experiments need to be performed to accelerate the clinical translation of these findings. Dipak Kumar Sahoodeng et al. described the effects of various natural antioxidant components on IBD (169). In the following section, we provide a summary of natural products that regulate UC by modulating mitochondrial dynamics and mitophagy.

**Table 1:** Effects of natural products on UC-related tissues through mitophagy.

Nature products	Species	Experimental models	Signaling pathway	Main results	Ref.
curcumin (50 mg/kg)	BALB/c mice	3.5%DSS induced	SIRT1/mTOR	mTOR&SIRT1 ↑	(170)
		N=80/4groups		Atg12, Beclin-1&LC3-II ↓	
curcumin (200 mg/kg)	IPEC-J2	H2O2 induced N=24/4groups	AMPK/TFEB/pink1/parkin	Pink-1&and Parkin ↑	(171)
				LC3-II&Beclin1 ↑	
				BNIP3L, BNIP3and FUNDC-1	
curcumin (200 mg/kg)	piglets	diquat induced	P-ERK	PGC-αand NRF-1 ↑	(172)
		N=24/4groups		P-PERK/PERK and MFN2 ↓	
curcumin (15、30、60mg/kg)	BALB/c mice	5%DSSinduced&2.5% ethanol IP.	p38MAPK	p-p38MAPK ↑	(173)
		N=60/6groups			
curcumin (200 mg/kg)	BALB/c mice	3%DSS induced	NS	LC3-II/LC3-I and Beclin-1 ↑	(174)
		N=45/3groups		p62 ↓	
curcumin (15、30、60mg/kg)	BALB/c mice	3%DSS induced	NS	Bcl-2 ↑	(175)
		N=40/5groups		Atg5, LC-3IIand Beclin-1 ↓	
RES (80 mg/kg)	BALB/c mice	3.5%DSS induced	SIRT1/mTOR	mTOR&SIRT1 ↑	(170)
		N=80/4groups		Atg12, Beclin-1and LC3II ↓	
RES (100 mg/kg)	piglets	diquat induced	pink1/parkin	Pink1and Parkin ↑	(176)
		N=24/4groups		LC3II and LC3- II/LC3- I ↑	
RES (100 mg/kg)	C57BL/6 mice	3%DSS induced	NS	LC3B, Beclin-1and LC3-II/I ↑	(177)
		N=48/4groups			
RES (100 mg/kg)	C57BL/6 mice	3%DSS induced	AMPK	CDX2, p-AMPKand SIRT1 ↑	(178)
		N=50/5group		p- NF-κB ↓	
Berberine (25、50、100mg/kg)	C57BL/6 mice	DSS induced and LPS-induced RAW264.7 cells	IRGM1/PI3K/ AKT/mTOR	IRGM1 ↑	(179)
		N=105/7groups		p-mTOR, p-AKT&p-PI3K ↓	
Berberine (25、50、100mg/kg)	KuNSing mice	3%DSS induced N=120/6groups	AMPK/mTOR/ULK1 ATG16L1/NOD1/RIPK2	p-ampk and p-ulk1 ↑	(180)
				LC3B, ATG12and ATG16L1 ↑	
				p- mtor, NOD1and RIPK2 ↓	
Baicalin (1、5 、10μ g/mL)	HT-29 (Human)	LPS induced	NS	LC3, Atg5 and BECN1 ↑	(181)
Ginsenoside Rd (10、20、40mg/kg)	C57BL/6 mice	DSS induced	AMPK/ULK1	p62, AMPK and p-ULK1 ↑	(182)
	THP-1	LPS + ATP induced			
Ginsenoside Rh2 (50mg/kg)	C57BL/6 mice	3%DSS induced	STAT3/miR-214	PTEN ↑	(183)
		N=24/4groups		p-STAT3and miR- 214↓	
Ginsenoside Rk2 (5、10、20μM)	THP-1	LPS induced	SIRT1/ERK/MEK	SIRT1 ↑	(184)
				P- ERK, MEK and SIRT1 ↑	

NS, Not specified; HT-29, The human colonic epithelial cell line;THP-1, human intestinal epithelial THP-1 cells. ↑, up; ↓, down.

#### 6.2.1 Curcumin with mitophagy on UC

Curcumin is the main active ingredient in dried, powdered rhizome of turmeric and has been found to exert beneficial effects against the development and progression of UC in animal and human trials (185, 186). A meta-analysis on the use of curcumin to treat UC comprising nine randomized controlled trials found that compared with the control group, the curcumin treatment increased the clinical remission rates (RR=2.28, 95%CI[1.43, 3.62], P=0.0005) and endoscopic remission rates (RR=1.66, 95%CI[1.07, 2.60], P=0.03), without inducing significant adverse events (187). Another study showed that it enhanced G2/M cell cycle arrest and autophagy and low doses of curcumin may activate adaptive stress responses, while high doses trigger acute responses (188). Shuting Cao et al. demonstrated that H_2_O_2_-induced epithelial barrier disruption and mitochondrial dysfunction models of UC in IPEC-J2 cells, curcumin treatment upregulated Pink1 and Parkin genes and proteins, but did not affect the expression of Nix, BNIP3, and FUNDC-1, necessitating the hypothesis that curcumin may activate mitophagy primarily via the Pink1-Parkin pathway (171). Other studies have demonstrated that curcumin abolished the diquat-induced oxidative stress and jejunal injury in piglets, enhanced the activity of complexes I-IV and suppressed the expression level of phosphorylated-PERK/PERK and phosphorylated-eIF2α/eIF2α, to improve the expression of mitochondrial function (172).

#### 6.2.2 Resveratrol with mitophagy on UC

The RES is a natural polyphenol found in various plants and fruits (189). Dietary supplementation of RES improved intestinal barrier integrity, oxidative stress, and intestinal inflammation in a colitis model (190). The study by Shuting Cao et al. concluded that RES activated mitophagy which was indicated by the upregulated expression of Pink1, Parkin, and LC3-II/LC3-I relative to the piglets injected with diquat (176).

#### 6.2.3 Berberine with mitophagy on UC

Berberine is a quaternary ammonium alkaloid primarily extracted from Coptis chinensis and Phellodendron amurense (191). It has the potential to treat various diseases such as cardiac aging and acute kidney injury via enhancing mitophagy (192, 193). Approximately, 211 potential targets of Berberine and 210 UC genes were predicted on the PharmMapper database whereas UC genes were determined on the GeneCards database and the OMIM database (194). Berberine targets IRGM1 to inhibit the PI3K/AKT/mTOR pathway, suppressing inflammatory response in UC (179). Similarly, Berberine was found to stimulate autophagy via the AMPK/MTOR/ULK1 pathway, as well as inhibit lysozyme and its secretion, to accelerate lysosomal maturation and expression, implying that it has the potential to treat inflammation (180).

#### 6.2.4 Ginsenoside with mitophagy on UC

Ginsenoside is the most abundant active ingredient in the traditional Chinese medicine ginseng, with diverse structures. Evidence for its anti-fatigue, immunomodulation, and anti-tumor properties have been documented (195). In a meta-analysis comprising 15 studies with 300 animals, it was observed that ginsenosides significantly reduced the levels of pro-inflammatory factors (IL-β, IL-6, TNF-α) and upregulated the expression of the anti-inflammatory factors IL-10 and tight junction proteins (Zonula Occludens-1, occludin) (196). Elsewhere, oral administration of ginsenoside Rd alleviated the DSS-induced enteritis symptoms in a dose dependent-manner. *In vitro*, ginsenoside Rd significantly inhibited the NLRP3 inflammasome, enhancing the p62-dependent mitochondrial translocation and mitophagy predominantly through the AMPK/ULK1 signaling pathway (182).

These natural compounds have shown promising potential in treating UC. The cellular and animal models discussed in the above sections have uncovered the pivotal roles of natural products in enhancing mitophagy via pink1/parkin, AMPK/mTOR/ULK1, and SIRT1/ERK/MEK pathways, to prevent inflammation and enhance the repair of intestinal barrier. However, overstimulation or inhibition of mitochondrial function may result in detrimental effects, and thus, it is imperative to balance between mitochondrial dynamics and mitophagy to achieve UC treatment. Several animal model experiments have explored the mechanisms of chronic UC, identifying new targets for drug development. Compared with pharmaceutical drugs, natural ingredients have the advantages of being multi-target and eliciting fewer side effects. However, the efficacy of such drugs is based on findings from preclinical investigations, necessitating further well-designed clinical trials to verify their clinical efficacy and address the current limitations.

## 7 Concluding remarks and future perspectives

In conclusion, a strong link exists between intestinal barrier damage, chronic inflammation, and mitochondrial dysfunction in ulcerative colitis. Natural products targeting these processes show promise as potential treatments. However, several challenges remain to be solved. 1) Although the roles of mitochondrial dynamics and autophagy in UC have been recognized, the precise regulation of the balance between mitochondrial fission and autophagy is poorly understood. In addition, several differences exist mitochondrial function and autophagic response across different species, making it difficult to formulate appropriate targeting strategies. Most of the current studies are based on animal models, with few studies conducted in actual UC patients. 2) Although several natural products have been found to modulate mitochondrial autophagy, most of such studies involved *in vitro* experiments and small-scale animal models. Therefore, the clinical efficacy of natural products need to be validated because data from preclinical trials may not accurately reflect the clinical settings, in terms of the dosage, safety, and side effects. 3) Current treatments for UC often target mitochondrial fission or autophagy, but these approaches may not be specific to individual patient needs. How to select appropriate therapeutic targets and avoid over-regulation of mitochondrial function remains to be clarified.

In the future, clinical trials for various mitochondria-targeted therapies should be conducted to confirm the therapeutic efficacy of natural products discussed in this review. The trials should integrate a wide spectrum of data, including clinical profiles, gene mutations, and gut molecular signatures, to identify patient subgroups that are most likely to benefit. Moreover, an integrated approach is essential to address the inherent heterogeneity of UC and enhance the application of personalized treatment. This primary focus of this review was to enhance the current understanding of the complexity of UC and discuss the available treatments for specific patient subgroups.
